# Prevalence of pediatric surgical problems among east African refugees: estimates from a cross-sectional survey using random cluster sampling

**DOI:** 10.1186/s12887-022-03576-9

**Published:** 2022-09-01

**Authors:** Zachary Obinna Enumah, Mohamed Yunus Rafiq, Daniel Rhee, Frank Manyama, Hilary Ngude, Kent Stevens, Omar Juma, Joseph V. Sakran

**Affiliations:** 1 Department of Surgery, Johns Hopkins Global Surgery Initiative (JHGSI), Johns Hopkins Hospital, Tower 110 Doctor’s Lounge, 600 N. Wolfe Street, Baltimore, MD 21287 USA; 2 Department of International Health, Johns Hopkins School of Public Health, Baltimore, MD USA; 3 Department of Anthropology, New York University Shanghai, Shanghai, China; 4 Ifakara Health Institute, Bagamoyo, Tanzania; 5 Tanzania Red Cross Society, Dar es Salaam, Tanzania

**Keywords:** Global surgery, Refugee health, Pediatrics, Tanzania, Conflict setting

## Abstract

**Importance:**

Surgery is a foundational aspect to high functioning health care systems. In the wake of the Lancet Commission on Global Surgery, previous research has focused on defining the burden of surgical conditions among a pediatric population, however these studies often fail to include forced migrant or refugees. The goal of this study was to estimate the prevalence of pediatric surgical conditions among refugees in east Africa.

**Methods:**

We used the previously validated Surgeons OverSeas Assessment of Surgical Need (SOSAS) that utilizes cross-sectional design with random cluster sampling to assess prevalence of surgical disease among participants aged 0 to 18 years in Nyarugusu refugee camp, Tanzania. We used descriptive and multivariable analyses including an average marginal effects model.

**Results:**

A total of 1,658 participants were included in the study. The mean age of our sample was 8.3 ± 5.8 years. A total of 841 participants (50.7%) were male and 817 participants (49.3%) were female. A total of 513 (*n* = 30.9%) reported a history or presence of a problem that may be surgical in nature, and 280 (54.6%) of them reported the problem was ongoing or untreated. Overall, 16.9% had an ongoing problem that may be amenable to surgery. We found that increasing age and recent illness were associated with having a surgical problem on both our multivariable analyses.

**Conclusion:**

To our knowledge, this is the first and largest study of prevalence of surgical conditions among refugee children in sub-Saharan Africa. We found that over 16% (one-in-six) of refugee children have a problem that may be amenable to surgery. Our results provide a benchmark upon which other studies in conflict or post-conflict zones with refugee or forced migrant populations may be compared.

## Introduction

Surgery is a cornerstone of any high functioning health care system globally [1]. It has been estimated that up to 28% of the global burden of disease may require surgical management at some point in time [2]. With accidents, trauma, and injury common among young persons, the estimates for children are even large, with up to 85% of children in low- and middle-income countries (LMICs) potentially having a condition amenable to surgical treatment by age 15 [3, 4]. Additionally, children have unique surgical and anesthetic needs with the prospect, though, of reversing years of disability or morbidity through intervention. In the wake of the Lancet Commission on Global Surgery’s, global surgery has gained increase attention during the last several years with increased attention on addressing the burden of disease, surgical infrastructure, and cost effectiveness [5–7]. With the enormous burden of surgery especially among children, some research has emerged focusing on the pediatric burden of surgical disease [8, 9]. Yet, still scarce is research focused on pediatric surgical conditions, and even more specifically among forced migrants, such as refugees.

Globally, there are over 25 million refugees, and over half of the world’s refugees are children. A large body of literature exists on the health of refugee children, but notably absent from this body of literature is a focus on surgery. Few studies do address pediatric burden of surgery to some degree, but most of these are small, hospital-based studies that fail to capture the disease burden among those for whom health care is unavailable or inaccessible [10, 11]. Or, these studies focus on refugees who have resettled to third countries [12]. The few that have provided population level estimates using household surveys do not focus on refugee children in a conflict or post-conflict zone [8, 9].

Using the previously validated Surgeons Overseas Assessment of Surgical Need (SOSAS) tool, the goal of this study was to estimate the prevalence of pediatric surgical conditions among refugees in east Africa and explore factors associated with having a surgical problem among children.

## Methods

### Study setting

We performed the study in Nyarugusu refugee camp, which is located in western Tanzania and home to approximately 130,000 refugees from the Democratic Republic of Congo and Burundi. The study took place in Nyarugusu refugee camp, located in Kigoma region of Tanzania. It is a protracted refugee situation, as it has been in continuous existence since 1996. The camp is divided into 14 zones that are further sub-divided into villages and clusters of varying sizes.

### Data collection

Data for this investigation is drawn from the parent study assessing the burden of surgical disease among refugees in western Tanzania where 1,658 met our inclusion criteria. In the parent study that had a 99% response rate, a total of 3,574 refugees were interviewed from 126 clusters (out of the initial randomly selected 132 clusters) [13]. The original required sample size for the parent study was calculated to be 3519 individuals based on the following equation: *n* = Z^2^p(1-p)/L^2^, where n is the total sample size, 95% Z value is 1.96, p is the estimated prevalence of surgical disease, and L is the accepted range around the estimated prevalence. We used a prevalence of 11% and an accepted range of 1.5% based on previous research, and we also used a design effect of two and accounted for 5% non-response [14]. Inclusion criteria included those aged 18 years of age or younger who consented for the study and were living in Nyarugusu refugee camp. The data comes from an adapted version of the Surgeons Overseas Assessment of Surgical Needs (SOSAS) tool [15]. The SOSAS tool has been utilized in several other LMICs to date including in pediatric populations and in one refugee population [16]. Using random cluster sampling, we randomly selected 132 clusters from a total of 1472 from our sampling framework based on the administrative divisions of zones, villages, clusters and households. All households within a cluster were approached, and two people within each household were randomly selected to participate in the survey. Data was collected in using REDCap Mobile in an offline fashion and subsequently uploaded daily to the secure Johns Hopkins University REDCap data server. Data was collected by refugee community health care workers who participated in a training workshop prior to the start of data collection.

### Data analysis and ethical approval

Stata 16 (Stata 16, College Station, TX.) was used for all data analysis. Descriptive analysis was performed using t-tests and Chi square tests, where continuous variables have been reported as means with standard deviation and categorical variables presented as total number and relevant percentages. Additionally, a p-value of less than 0.05 was used for statistical significance. We used multivariable logistic regression and an average marginal effects model to assess for associations between specific covariates and the history of presence of a surgical problem. We reported odds ratios and 95% confidence intervals. Standard errors were adjusted for the 126 clusters included in the final analysis. For our multivariable model, reference groups included residence in zone 3 (location of main hospital), age under 1, male sex, Congolese nationality, no education, illiteracy, poor health status, no primary health care utilization, and no illness within the past year. We used zone 3 as a reference group for our multivariable model in an effort to assess effect of distance to the main hospital on history or presence of a surgical problem. Zone 3 is where the main health center in the camp is located and where surgery is performed. The variables for the multivariable model were almost all significant on univariable analysis (e.g. *p* < 0.05) with the exception of nationality which has epidemiological importance. Religion was not included in the final model because it was not significant on univariable analysis, and almost all respondents were Christian.

In an effort to further evaluate the association between the covariates and the primary outcome of interest (history of presence of surgical problem), we used an average marginal effects model. The model works by calculating the predicted probability of the outcome based on the reference covariate (e.g. male sex), repeating the prediction by changing the covariate of interest to the value of interest (female sex), and computing a difference between the two (marginal effect). This was then repeated for all observations and then averaged to produce the average marginal effect of that covariate on predicted probability of a surgical problem. We repeated this for the covariates in the same model as the multivariable logistic regression. This was accomplished using the -margins and -marginsplot commands in Stata, including the production of the accompanying figure. Importantly, these estimates are an effect on probability—which is distinct from a calculated odds or odds ratio.

The study was approved by the Johns Hopkins Medicine Institutional Review Board (IRB00258009). Research clearance was also obtained from the Tanzanian Commission on Science and Technology (2020–391-NA-2011–143). A permit to enter the refugee camp was granted by the Tanzanian Ministry of Home Affairs. Informed consent/assent was obtained from all participants/their parents/guardians/adult member of the household.

## Results

Data collection occurred between August 4, 2021 and September 10, 2021. A total of 1,658 records were included in the study after excluding patients older than 18 years of age and those who did not consent to the study (*n* = 1) or for those for whom consent was missing (*n* = 3). We had a 99.8% response rate.

### Demographic profile

**Fig. 1:**
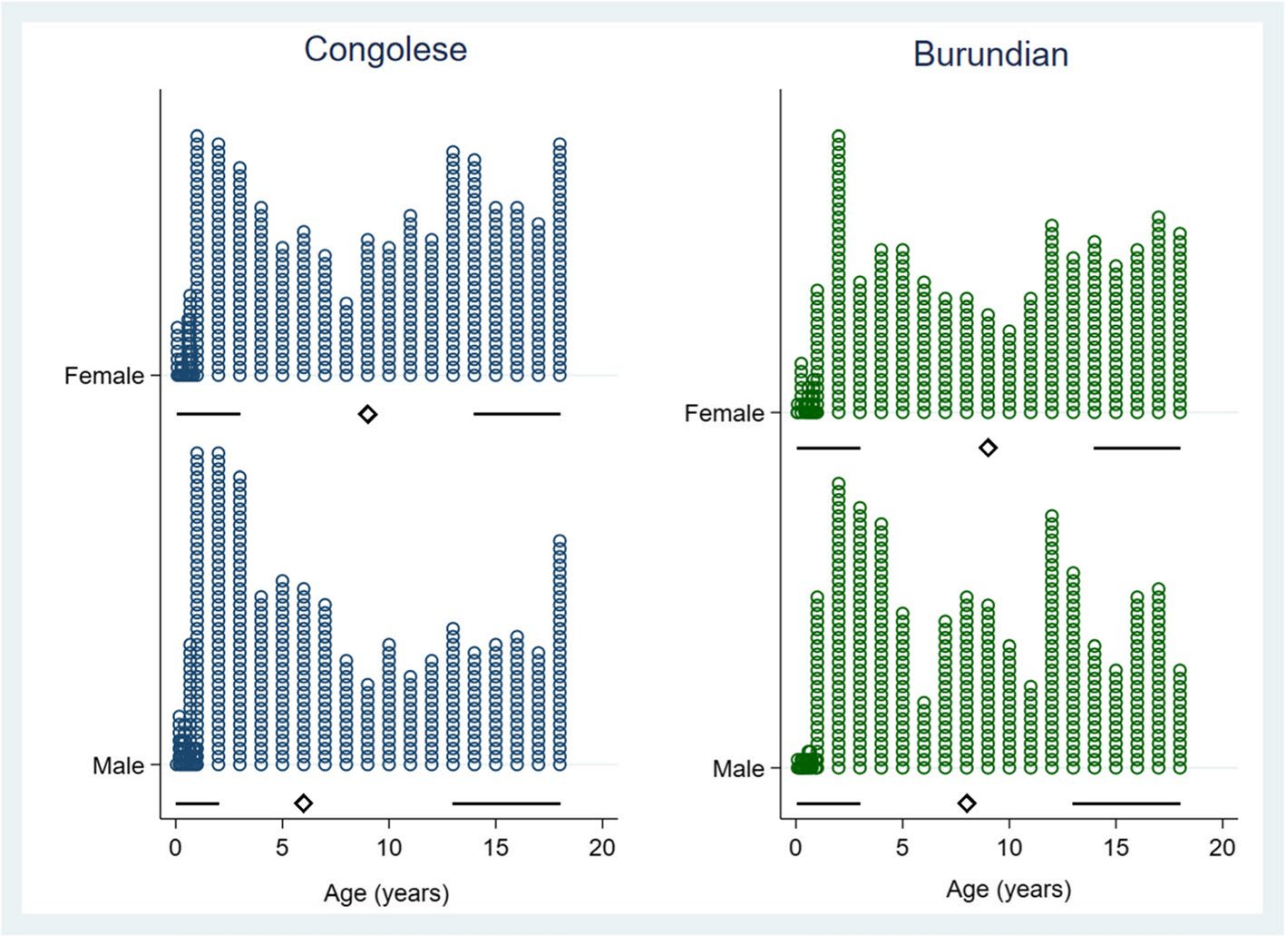
Age distribution of study population by sex and nationality. Underneath histogram, in the Tufte plot, diamonds represents median value, gaps represent interquartile range, and lines represent whiskers

The mean age of our sample was 8.3 ± 5.8 years, and the most populous group were those aged 12 to 18 years (*n* = 593, 35.8%), followed by those aged 1 to 5 (*n* = 543, 32.8%) (See Fig. 1). A total of 841 participants (50.7%) were male and 817 participants (49.3%) were female. There were approximately equal number of patients from the DRC (*n* = 870, 52.5%) and Burundi (*n* = 784, 47.3%) with 3 participants (0.2%) reporting another nationality. The most common level of education attained was no formal schooling (*n* = 750, 45.3%), followed by primary school (*n* = 671, 40.5%). Most participants were not employed (*n* = 1,366, 82.5%), unmarried (*n* = 1,362, 82.4%), and Christian (*n* = 1,571, 95.0%). Under half reported literacy (*n* = 722, 43.5%). See Table 1.

**Table 1:** Demographic profile of study population

	**Total**
	*N* = 1,658
**Age, mean ± SD**^a^	8.3 ± 5.8
**Age Category**	
Under 1	139 ( 8.4%)
Age 1 to 5	543 (32.8%)
Age 6 to 11	383 (23.1%)
Age 12 to 18	593 (35.8%)
**Sex**	
Male	841 (50.7%)
Female	817 (49.3%)
**Nationality**	
Congolese	870 (52.5%)
Burundian	784 (47.3%)
Other	3 ( 0.2%)
**Education**	
None	750 (45.3%)
Primary School	671 (40.5%)
Secondary School	235 (14.2%)
Higher Education (e.g. college)	1 ( 0.1%)
**Occupation**	
Unemployed	1,366 (82.5%)
Farmer	11 ( 0.7%)
Small Business	7 ( 0.4%)
Housemaid	2 ( 0.1%)
Self-employed	8 ( 0.5%)
Mother of the Home (Stay-at-home mother)	11 ( 0.7%)
Other	250 (15.1%)
**Marital Status**	
Married	45 ( 2.7%)
Single	1,362 (82.4%)
Divorced	3 ( 0.2%)
Other	243 (14.7%)
**Religion**	
Christian	1,571 (95.0%)
Muslim	62 ( 3.7%)
Other	21 ( 1.3%)
**Literate**	
No	936 (56.5%)
Yes	722 (43.5%)

^a^*SD* Standard deviation

### Health status and surgical pathology

The majority of participants reported generally good health (*n* = 1,398, 85.1%) and most reported utilizing the biomedical health services in the refugee camp (*n* = 1,524, 91.9%). Over half of all participants (*n* = 982, 59.3%) reported being sick within the last year with a median number (IQR) of weeks ill of 2 visits (1–3), and a median number (IQR) of health visits of 2 visits (1–3) within the past year. Of those who did reported being sick within the past year, most participants reported recovering from their illness (*n* = 838, 85.6%).

A total of 513 (*n* = 30.9%) reported a history or presence of a problem that may be surgical in nature. Of those who did report a problem, 280 (54.6%) reported the problem was ongoing or untreated. Of the total study population, 16.9% had an ongoing problem that may be amenable to surgery. Anatomical breakdown of problems included the face/head/neck (*n* = 247, 15.0%), chest/breast (*n* = 33, 2.09%), back (*n* = 25, 1.5%), abdomen (*n* = 99, 6.0%), groin (*n* = 75, 4.5%), and extremities (*n* = 182, 11.0%). See Table 2.

**Table 2:** Health status, surgical problems, and anatomical locations

	**Total**
	*N* = 1,658
**Are you generally healthy?**	
Yes	1,398 (85.1%)
No	244 (14.9%)
**Have you ever been to the clinic or hospital in the camp?**	
Yes	1,524/(91.9%)
No	134 ( 8.1%)
**Sick within the last year?**	
Yes	982/1,656 (59.3%)
No	674/1,656 (40.7%)
**Time ill (weeks), median (IQR)**^a^	2 (1–3)
**Number of visits to health center, median (IQR)**^a^	2 (1–3)
**Recovered from illness?**^a^	
Yes	838 (85.6%)
No	141 (14.4%)
Surgical/Anatomical Area	
**Face/Head/Neck**	247 (15.0%)
**Chest/Breast**	33 ( 2.0%)
**Back**	25 ( 1.5%)
**Abdomen**	99 ( 6.0%)
**Groin**	75 ( 4.5%)
**Extremities**	182 (11.0%)
**Any surgical problem**	513 (30.9%)
**Ongoing Problem**^b^	280(54.6%)

^a^Denominator is among those who reported being sick within the last year

^b^Denominator for ongoing surgical problem is among those who reported history of presence of any surgical problem (*n* = 513)

### Multivariable analysis and average marginal effects model

**Fig. 2:**
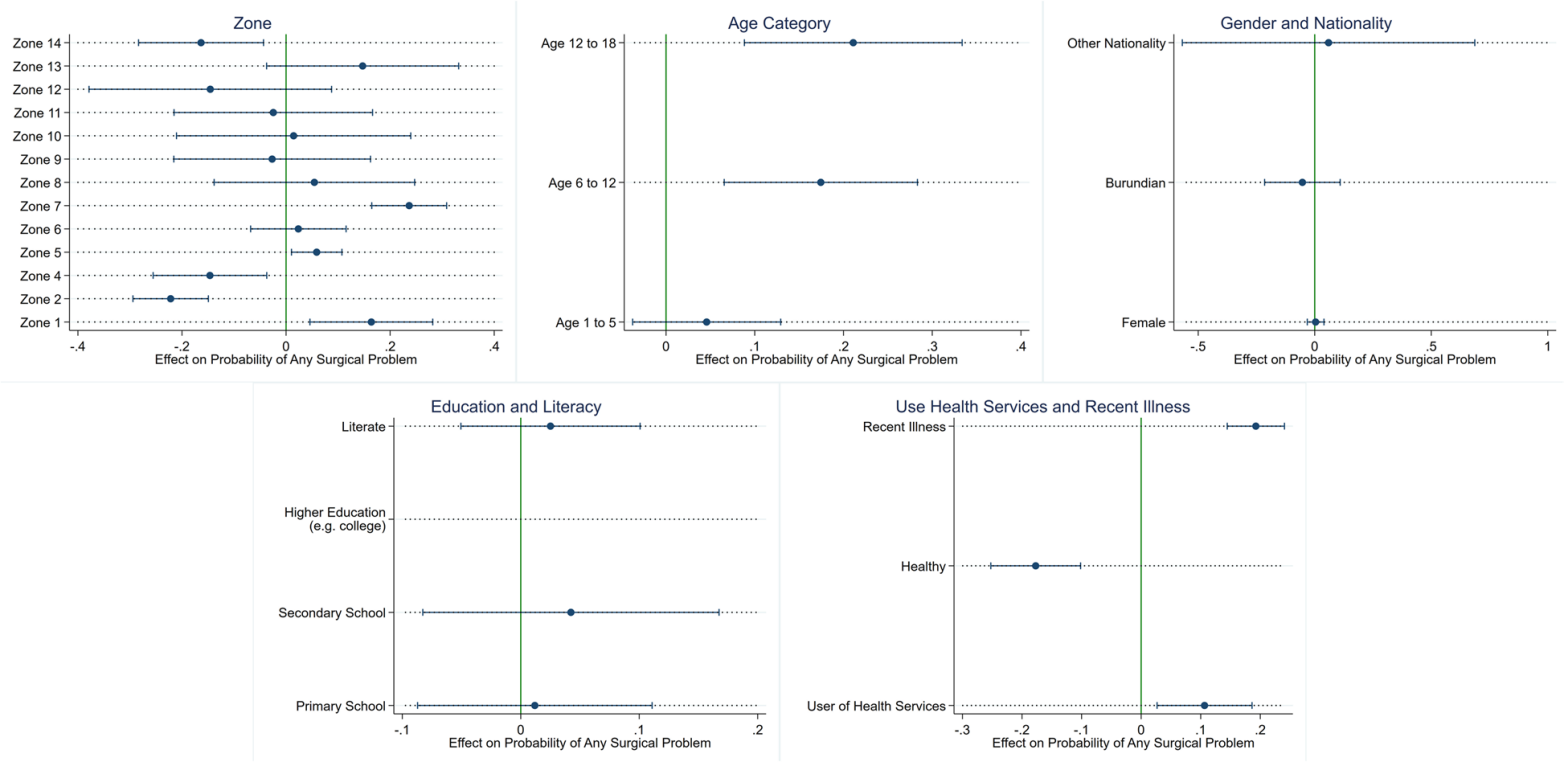
Average marginal effects model on covariate effect on probability of any surgical problem. Null value of effect on probability is 0. If confidence interval crosses this null value, the result was not significant

Results of the multivariable regression model are displayed in Table 3. On multivariable analysis, age 6 to 11 (OR = 2.96, 95% CI 1.37 to 6.38) and age 12 to 18 (OR = 3.57, 95% CI 1.55 to 8.21), use of health services (OR = 1.96, 95% CI 1.12 to 3.41), illness within the past year (OR = 3.08, 95% CI 2.28 to 4.15), and zone were associated with having a surgical problem (See Table 3). Being healthy was associated with a 61% decreased odds of having a surgical problem (OR = 0.39, 95% CI 0.26 to 0.57). There was no association between age 1 to 5, female sex, nationality, education, or literacy on having a surgical problem. Findings from the average marginal effects model confirmed that of the multivariable logistic regression model. Age categories of age 6 to 11 and age 12 to 18 were both significantly associated with increased probability of having a surgical problem. Similarly, use of primary health services, illness in the past year, and certain zones were associated with increased probability of having a surgical problem (see Table 3, Fig. 2).

**Table 3:** Multivariable analysis and average marginal effects model of factors associated with having any surgical problem among a pediatric population

	**Multivariable**^a^		**Average Marginal Effects**^b^	
**Covariates**	**Odds Ratio**	**95% Confidence Interval**	**dy/dx**	**95% Confidence Interval**
**Zone**				
1	2.27	1.29 – 3.99	0.16	0.05 – 0.28
2	0.19	0.11 – 0.33	-0.22	-0.29—-0.15
3	REF	–	REF	–
4	0.40	0.18 – 0.88	-0.15	-0.26—-0.04
5	1.36	1.06 – 1.74	0.06	0.01 – 0.11
6	1.13	0.70 – 1.83	0.02	-0.07 – 0.12
7	3.22	2.29 – 4.51	0.24	0.16 – 0.31
8	1.33	0.49 – 3.62	0.05	-0.14 – 0.25
9	0.86	0.30 – 2.45	-0.03	-0.22 – 0.16
10	1.08	0.33 – 3.57	0.01	-0.21 – 0.24
11	0.87	0.31 – 2.50	-0.02	-0.22 – 0.17
12	0.40	0.07 – 2.16	-0.15	-0.38 – 0.09
13	2.09	0.81 – 5.37	0.15	-0.04 – 0.33
14	0.35	0.13 – 0.92	-0.16	-0.28—-0.04
**Age**				
Under 1	REF	–	REF	–
Age 1 to 5	1.39	0.73 – 2.64	0.05	-0.04 – 0.13
Age 6 to 11	2.96	1.37 – 6.38	0.17	0.07 – 0.28
Age 12 to 18	3.57	1.55 – 8.21	0.21	0.09 – 0.33
**Sex**				
Male	REF	–	REF	–
Female	1.02	0.83 – 1.26	0.004	-0.03 – 0.04
**Nationality**				
Congolese	REF	–	REF	–
Burundian	0.74	0.29 – 1.89	-0.05	-0.21 – 0.11
Other	1.38	0.05 – 38.85	0.06	-0.57 – 0.69
**Education**				
No education	REF	–	REF	–
Primary School	1.07	0.60 – 1.91	0.01	-0.09 – 0.11
Secondary School	1.27	0.63 – 2.57	0.04	-0.08 – 0.17
Higher Education^c^	–	–	–	–
**Literate**				
No	REF	–	REF	–
Yes	1.16	0.75 – 1.79	0.03	-0.05 – 0.10
**Healthy?**				
No	REF	–	REF	–
Yes	0.39	0.26 – 0.57	-0.18	-0.25—-0.10
**Use PHC**				
No	REF	–	REF	–
Yes	1.96	1.12 – 3.41	0.11	0.03 – 0.19
**Illness Past Year**				
No	REF	–	REF	–
Yes	3.08	2.28 – 4.15	0.19	0.14 – 0.24

Multivariable and average marginal effects models each include 1,623 observations. Abbreviations: *PHC* Primary health care

^a^Null value for confidence intervals for odds ratios is 1

^b^Null value for confidence intervals for average marginal effect is 0

^c^Only one individual reported tertiary education

## Discussion

To our knowledge, this is the first and largest study assessing underlying prevalence of surgical disease among a pediatric refugee population in sub-Saharan Africa. Other studies have focused on non-refugee populations or utilized hospital-based records to examine or estimate the burden of disease [8, 11]. Strengths of our study included a high response rate, cluster randomized design, and large sample size. Our study is not without limitations, though.

One limitation was that we did not confirm the history of presence of a surgical problem using physical examination by a trained physician or surgeon. This would have required extensive resources. Nevertheless, recent literature using the SOSAS tool has suggested a high correlation between the verbal head to toe examination and physical examination [14]. Still, though, this precluded our ability to potentially capture all surgical problems that may be in line with other studies (e.g. hernias, hydroceles), as the ones most reported in this study were perhaps more readily apparent—such as face/head/neck and extremities. Similarly, in order to capture as many cases as possible that may be surgical, participants were asked if they had a wound, burn, swelling, congenital or acquired deformity, or other question based on anatomical region (e.g. history of caesarean section, abdominal distension, operation on the face or neck) to ascertain if someone may have a problem that would potentially be amenable to surgery. While many of the above symptoms and problems may be amenable to minor or major surgery, there is a possibility an individual’s problem may not be operable and thus could overestimate the potential burden of conditions truly amenable to surgery. In spite of the above limitations, our findings do point to several key messages regarding the pediatric burden of surgical disease among refugees.

First, the untreated burden of surgical problems among refugee children and adolescents is high. Among children in non-refugee settings, other research has suggested high burden and prevalence of surgical problems from 3.5% to 19% [8, 9, 17–20]. Our estimates of over build on this data, where in our study population, over 30% reported a history or presence of a surgical problem, and of that 30%, over half (54%) reported the problem was ongoing. Extrapolating this number (16%) of the total study population that have an unmet surgical need to the entire 2,600,000 population of refugee children in the eastern and Horn of Africa region would result in over 400,000 operations needed [21]. Further, extrapolating this number to the entire approximately 13,000,000 refugee children would suggest a need of over 2,000,000 surgeries among refugees (not including other forced migrants, such as internally displaced persons) [22].

Other studies on surgical need among forced migrants have often focused on hospital-based data [11, 23, 24]. Here, we provide household level data suggesting a large unmet need of surgery among this refugee population with a finding that increasing age and recent illness were associated with having a surgical problem on both our multivariable and average marginal effects models. Part of this persistent need may be related to a lack of sufficient resources, including human resources, in addressing the surgical burden of disease, especially in conflict or post-conflict zones. In Tanzania, where refugees have been seeking protection for over six decades, there are relatively few pediatric surgeons and most who do specialize in it have historically obtained their training abroad [25]. In effect, many pediatric surgical conditions are managed by a general surgeon or general practitioner. In the context of this refugee camp, there is no formally trained surgeon, so surgeries are done almost exclusively by general practitioners with the exception of infrequent visiting medical missions (including co-authors ZOE and KAS). Additionally, there is currently no functioning general anesthetic machine, limiting surgeries to those which can be performed primarily under spinal anesthesia or without the need to intubate a patient. Many patients with surgical conditions are referred to other centers for surgery, but this process can be arduous and also may partly explain a high burden of unmet need in this refugee camp given long waits or delays for referral [26].

On a larger scale, countries such as Tanzania, have developed National Surgical Obstetric and Anesthesia Plans (NSOAPs) as a means by which to address core and foundational aspects of health care delivery and scale-up, yet a surprisingly low number of national health service plans even include language on pediatric surgery [27, 28]. As a response to the 2015 call to action by Dr. Jim Yong Kim of the World Bank that surgery is an “indivisible, indispensable part of health care,” the Tanzanian NSOAP was Launched in March 2018 by the Ministry of Health, Community Development, Gender, Elderly and Children [29]. This landmark plan by Tanzania included specific language focused on addressing the pediatric burden of surgical disease in Tanzania, including training 15 pediatric surgeons by 2025. Since refugee populations in Tanzania access national health care services, especially for advanced care that cannot be obtained in the refugee camp, understanding the trajectory of pediatric surgery in Tanzania is paramount to addressing the burden of surgical disease among forced migrants. Similarly, the convening of the Global Initiative for Children’s Surgery (GCIS) has brought increased attention to the importance of infrastructure, service delivery, training, research and partnerships between and among LMICs and high-income countries [30].

Common conditions among pediatric patients often include umbilical hernias, groin hernias, hydroceles, wound and burns. Many or all of these conditions should be managed at the level of the district hospital in Tanzania, and recent research has suggested that task shifting could play an important role in addressing this burden [31, 32]. Since physicians in humanitarian settings may not receive specialty training in surgery but are general practitioners, there may be an increased role for task shifting in such settings to help curb the burden of pediatric surgical disease. While our study did not assess emergency surgery needs, the same training may be said of appropriately training physicians, nursing and other health care staff in managing and stabilizing patients requiring pediatric emergency surgery, as this patient population has had high mortality in Tanzania [25]. In addition, improved referral networks may be needed to help manage a large burden of surgical disease. Refugees in Tanzania are legally not allowed to leave the camp without special permission or permit, and this may have contributed to a higher burden of unmet conditions if care is not available in the refugee camp and the referral process is delayed.

Our results provide a benchmark upon which other studies in conflict or post-conflict zones with refugee or forced migrant populations may be compared in assessing an unmet need of surgical disease. As we look to the future of global surgery, there is a need for continued development of robust capacity to study the epidemiology, demography, cost effectiveness, and outcomes of surgical disease in this specific population, while simultaneously working towards improving clinical capacity building. It is clearly evident that much work remains to be done to achieve the sustainable development goals and work towards “surgery and health for all” [33]. Targeting the burden of pediatric surgery is paramount in preparing for the health of the next generation. An increased focus on all of these efforts including infrastructure building, training, and research can be the multifaceted approach needed to better build capacity in both conflict and non-conflict settings.

## Data Availability

Data used and analyzed in the current study are not publicly available due to privacy and personally identifiable health information. De-identified, aggregate data is potentially available from corresponding author upon request.
